# Morphology of the Calcaneofibular Ligament Reflects Degeneration of the Talonavicular Articular Surface: A Cadaver Study

**DOI:** 10.3390/jcm13247565

**Published:** 2024-12-12

**Authors:** Ryuta Tanaka, Daisuke Kiyoshima, Kaori Suyama, Ning Qu, Miyu Inagawa, Shogo Hayashi

**Affiliations:** 1 Department of Anatomy, Division of Basic Medicine, Tokai University School of Medicine, Isehara 259-1193, Kanagawa, Japan; ryuta-tanaka@kantoh.johas.go.jp (R.T.); suyama@tokai.ac.jp (K.S.); sho5-884@umin.ac.jp (S.H.); 2 Central Department of Rehabilitation Medicine, Kanto Rosai Hospital, Kawasaki 211-8510, Kanagawa, Japan; 3 Department of Immunoregulation, Institute of Medical Science, Tokyo Medical University, Shinjuku, Tokyo 160-8402, Japan; quning@tokyo-med.ac.jp; 4 Department of Sports Orthopedic Surgery, Kanto Rosai Hospital, Kawasaki 211-8510, Kanagawa, Japan; i.3you3you@gmail.com

**Keywords:** ankle, calcaneofibular ligament, cartilage damage, osteoarthritis, chronic ankle instability, hindfoot motion

## Abstract

**Background:** Osteoarthritis is caused by damage to the articular cartilage due to bone-on-bone collisions and friction. The length, width, and thickness of the ligaments are expected to change in order to regulate excessive bone-to-bone movement. We aimed to clarify the relationship between ligament morphology and joint surface degeneration in the ankle joints using macroscopic observations and measurements. **Methods:** The participants were 50 feet of 45 Japanese cadavers. The lengths, widths, and thicknesses of the tibionavicular, tibiospring, tibiocalcaneal, posterior tibiotalar, anterior tibiotalar, and calcaneofibular ligaments (CFLs) were measured. The degeneration of the talonavicular joint surface was investigated macroscopically and classified into two groups: the Degeneration (+) group and Degeneration (−) group. Unpaired t-tests were performed for each measurement. Logistic regression analysis was performed on the significantly different items to obtain cutoff values, sensitivity, and specificity. **Results:** Only the width of the CFL differed significantly between the Degeneration (+) (20 feet) and Degeneration (−) groups (*p* < 0.001). In the logistic regression analysis, the width of the CFL had an R2 of 0.262, sensitivity of 75.0%, and specificity of 83.3%, with a cutoff value of 8.7 mm. **Conclusions:** A wide CFL indicates a high likelihood of talonavicular articular surface degeneration.

## 1. Introduction

Cartilage damage to the articular surface of the talus in the ankle joint occurs as a result of repeated trauma from sports activities, traffic accidents, and microtrauma in daily life [1,2,3,4]. These injuries have a significant impact on the individual’s daily life [4,5]. Articular cartilage is not capable of self-repair [6], and cartilage degeneration spreads from the injured area, leading to osteoarthritis [7]. In the ankle joint, the cartilage of the talus is weaker than that of the tibia, and blood flow to the subchondral bone is poor, resulting in most cases of damage occurring on the talus side [8]. One symptom of osteoarthritis is joint instability [9]. Ligaments, joint capsules, and musculature contribute to joint stability. The collateral ligaments of the ankle joint contribute to ankle joint stability and are broadly classified into the medially located deltoid (DL) and lateral ligaments (LLs) [10]. The DL regulates the internal shift of the center of body weight, the abduction and rotation of the foot, and the accompanying motion of the lower leg in the direction of internal rotation [11]. The LL regulates the outward shift of the center of body weight, the foot’s abduction and external rotation, and the accompanying external rotation of the lower leg [12]. It has been documented that magnetic resonance imaging (MRI) reveals evidence of cartilage damage following an injury to the lateral ankle ligament [13]. Furthermore, the proximal attachment area of the deltoid ligament, which does not exhibit cartilage damage, is observed to be wider in ankle joints that present cartilage damage [14]. However, the precise correlation between the presence or absence of degenerative changes in the talocrural articular surface and the morphology of the ankle joint ligaments remains unclear. Should a correlation be identified between the ankle ligament morphology and the degeneration of the talocrural articular surface, the screening of specific ligaments could prove an effective method of preventing serious cartilage damage. In light of the aforementioned hypothesis, which posits a correlation between ankle joint morphology and talocrural facet degeneration, this study sought to elucidate the relationship between talocrural facet degeneration and ankle joint ligament morphology.

## 2. Materials and Methods

### 2.1. Participants

We examined 50 feet (25 right and 25 left) of 45 Japanese cadavers fixed in 10% formaldehyde donated to the Tokai University School of Medicine between April 2020 and August 2023. The mean age of the cadavers was 88.1 ± 6.5 years (21 males, 23 feet; 24 females, 27 feet). The exclusion criteria were defined as ankle joints that exhibited no defects in the extremity and were suitable for dissection and measurement. The present study did not exclude any ankles.

### 2.2. Dissection Method

The fiber bundles of the ligaments were identified by their origin and insertion according to previous anatomical reports [15,16,17,18]. The DL was dissected from the fascia, capsule, tendons, fat, and connective tissue in the posterior portion of the medial skin. The anterior portion of the DL was dissected from the superficial to the deep portion and from the medial malleolus to the insertions to the navicular, cuneiform, and talar bones. The flexor digitorum longus and posterior tibialis tendons were removed to identify the posterior tibiotalar ligament (PTTL). The anterior talofibular (ATFL) and calcaneofibular ligaments (CFL) were carefully dissected to identify their origins and insertions. Finally, these ligaments were detached and the articular surface of the talonavicular joint was exposed to determine whether the cartilage in each talus was damaged.

### 2.3. Measurements of Ankle Ligaments

**Figure 1:**
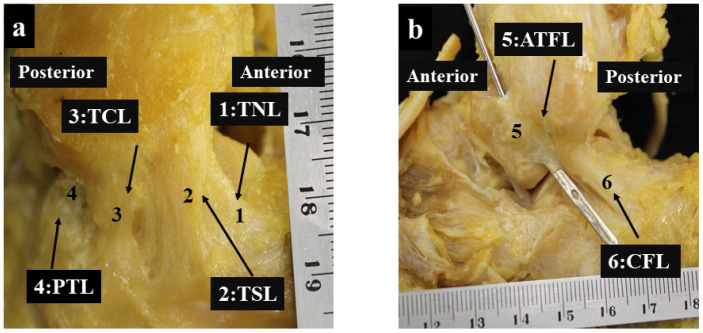
Measurements of each ankle ligament: (**a**) Deltoid ligaments measured in this study: 1. TNL, tibionavicular ligament, 2. TSL, tibiospring ligament, 3. TCL, tibiocalcaneal ligament, and 4. PTTL, superficial posterior tibiotalar ligament. (**b**) Lateral ligaments measured in this study were as follows: 5. ATFL, anterior talofibular ligament, and 6. CFL, calcaneofibular ligament.

The lengths, widths, and thicknesses of the DL, tibionavicular ligament, tibiospring ligament, tibiocalcaneal ligament, and PTTL were measured. Each ligament was carefully detached and measured using an electronic caliper equipped with a digital display (Shinwa Rules Corp., Niigata, Japan). The ligament length was defined as the length from the most distal to the most proximal part of the attachment. Width and thickness were measured at the ligament midpoint. For ATFL with two fiber bundles [15,16], which were found in seven feet, the length and thickness were calculated using each bundle, and the width was considered as their sum (Figure 1a,b).

### 2.4. Articular Surface Degeneration

The soft tissues associated with the ankle joint were removed, and the presence or absence of articular surface degeneration was macroscopically confirmed. Based on a study by Hirose et al. [19], joint surface degeneration was classified into four grades: grade 1, no pathological findings; grade 2, swelling or fibrosis; grade 3, fissures or clear erosion; and grade 4, cartilage loss. In this study, grade 2 or higher was defined as degeneration. The location of degeneration was classified as medial or lateral, based on the midpoint of the posterior margin of the joint.

### 2.5. Relationship Between the Presence of Cartilage Damage and Ligament Morphology

The ligaments were classified into the Degeneration (+) and Degeneration (−) groups. The length, width, and thickness of each ligament were compared between the two groups. All statistical analyses were performed using SPSS version 25.0 (IBM Corp., Armonk, NY, USA). Differences in mean values for the length, width, and thickness of each ligament were compared between the Degeneration (+) and Degeneration (−) groups using an unpaired *t*-test. Significance was set at *p* < 5%. Correlations were investigated using a logistic regression analysis. The cutoff values, sensitivity, specificity, and area under the curve were calculated from the receiver operating characteristic (ROC) curve.

### 2.6. Ethics

This study was conducted in accordance with the research index of the Japanese Society of Anatomists. The cadavers used in this study were donated to Tokai Daigaku Kentai No Kai for educational and research purposes. Informed consent was obtained from the ante-mortem person. This study was approved by the Ethics Committee of Tokai University School of Medicine (Ethics Review No. 19R-306).

## 3. Results

### 3.1. Articular Surface Degeneration

**Figure 2:**
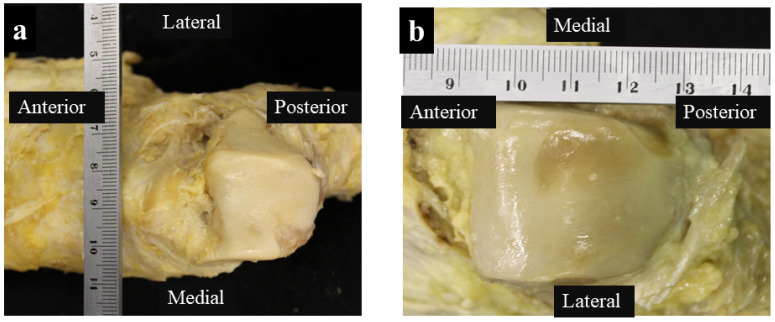
Cases of degeneration of the talocrural articular surface: (**a**) diagram showing the degeneration of the medial grade 4 articular surface; and (**b**) diagram of an ankle joint with grade 3 and 2 joint degeneration on the medial and lateral sides, respectively.

The degeneration of the talonavicular joint surface was observed in 20 of the 50 feet. Degeneration was found on the medial side of all 20 feet and on the lateral side of eight feet. No foot was found in which only the lateral side was degenerated (Table 1, Figure 2a,b).

### 3.2. Relationship Between the Presence of Cartilage Damage and Ligament Morphology

**Figure 3:**
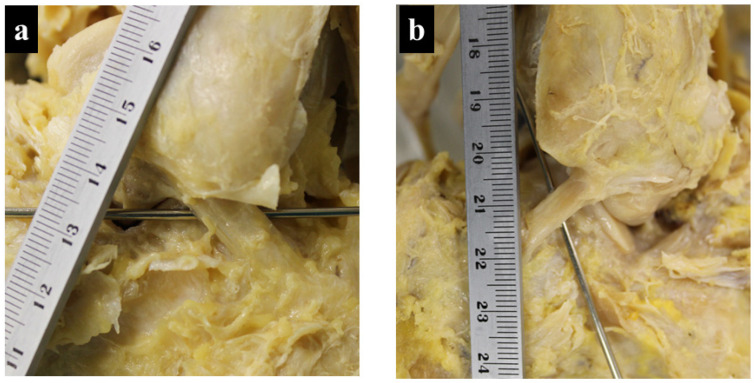
Cases with wide and narrow CFL observed in this study: (**a**) CFL width with talar articular surface degeneration; and (**b**) CFL width in the absence of talar surface degeneration. CFL: calcaneofibular ligament.

**Figure 4:**
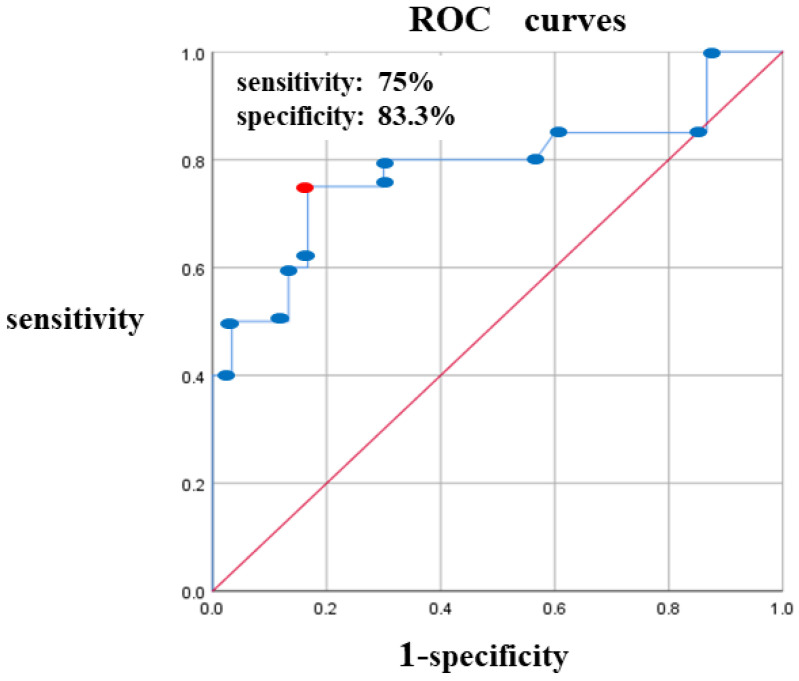
ROC curves for determining CFL width cutoff value, sensitivity, and specificity. The sensitivity and specificity were 75% and 83.3%, respectively, and the cutoff value for CFL width calculated from the ROC curve was 8.7 mm. CFL, calcaneofibular ligament; ROC, receiver operating characteristic.

A comparison between the Degeneration (+) and Degeneration (−) groups showed significant differences in the width of the CFL (*p* < 0.001), with 9.74 ± 2.48 mm in the Degeneration (+) and 7.38 ± 1.57 mm in the Degeneration (−) groups. For the other measurement items, no significant differences were observed between the two groups (Table 2, Figure 3a,b). Logistic regression analysis revealed a correlation coefficient of R^2^ = 0.262 (*p* < 0.001). The cutoff value was 8.7 mm, sensitivity was 75%, specificity was 83.3%, and area under curve was 78.4% (Figure 4, Table 3).

### 3.3. Availability of Ultrasound Imaging Device for the Target Ligament

**Figure 5:**
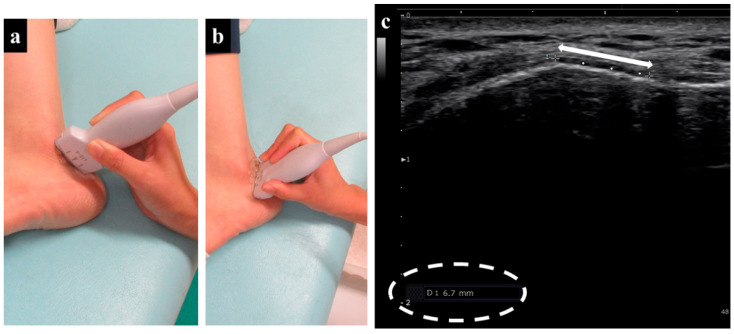
Method used to measure the width of the CFL using ultrasound imaging equipment: (**a**) after palpation, the CFL is extracted from the fibula to the talus in the long axial direction; (**b**) CFL width was measured by placing it in the short-axis direction at the midpoint; and (**c**) width measured in the short-axis image (6.7 mm). CFL: calcaneofibular ligament.

The left foot of one author (RT) was examined using an ultrasound imaging system (SONIMAGE HS2; Konica Minolta, Tokyo, Japan) to verify whether the CFL could be imaged and measured. After identifying the CFL by palpation, the probe was placed along the long axis of the ligament. The CFL was imaged between the fibula and calcaneus (Figure 5a). The probe was set at the midpoint of the CFL along the short axis (Figure 5b), and the width of the ligament was measured (Figure 5c).

## 4. Discussion

These results suggest that the CFL widens when articular surface degeneration is present. Berndt et al. [1] reported that the posterior medial aspect of the talar pulley impacted the tibial canopy when the ankle was forced medially during plantar flexion, whereas the lateral anterior aspect of the talar pulley impacted the fibula when the ankle was forced medially during dorsiflexion. Flick et al. [2] reported that osteochondral injuries on the lateral and medial sides of the talar pulley were associated with a history of trauma. However, the detailed pathogenesis of these injuries remains unclear, and their treatment is difficult and problematic [20,21,22,23]. To date, the LL has been reported to be damaged by trauma. Although cartilage damage after injury to the LL has been reported using magnetic resonance imaging [13], studies are lacking on the morphology of ankle joint ligaments with talar cartilage damage or on the evaluation of the morphology of the talonavicular surface and ligament with the naked eye. In the present study, the only significant difference found between the Degeneration (+) and Degeneration (−) groups was the width of the CFL, which mainly regulates the internal motion of the foot. A previous study reported that foot alignment in ankle osteoarthritis often occurs in an adducted position at the end of the disease on radiographic evaluation [24].

Similar to this study, in which all feet were found to have articular degeneration, many cases of medial damage to the articular cartilage have also been reported [2,21]. Furthermore, ankle joint instability and talar cartilage injury are more likely to occur in patients with a history of medial sprain, who tend to damage the LL [9,25,26]. Patients with ankle instability have a greater angle of foot medial rotation during gait than normal individuals [27,28], and a greater medial load to the talocrural joint surface [29]. Repeated microdamage is reported to thicken ligaments due to mechanical stress [30,31], and the ligament is reported to be larger in the area of loading as a result of joint instability [32]. Although the possibility that the CFL was originally wider in the degenerative group cannot be denied, the CFL might have become wider as a result of greater-than-normal secondary microstresses.

The effects of talar cartilage lesions can occur from the acute to the chronic phase, especially after foot ligament injuries, regardless of age [3,33,34]. Therefore, preventive intervention at an early stage is necessary. Kandel et al. [35] reported that ultrasound imaging devices could be used to measure the ligament morphology. Cao et al. [36] reported that ultrasound imaging devices are useful for diagnosing ankle ligaments in terms of both sensitivity and specificity. To examine the feasibility of this study in actual clinical situations, we measured the width of the ankle joint ligaments using an ultrasound imaging device. The CFL width was easily measured using ultrasound imaging. We believe that the CFL width can be evaluated over time and screened using ultrasound imaging, which could be a method for the early detection of foot osteoarthritis in each age group. The results of this study revealed that talonavicular facet degeneration was strongly related to the CFL width. Furthermore, we showed that the measurement of the CFL width using ultrasound imaging may be a screening tool for understanding the condition of the articular surface.

The limitations of this study are as follows: the mechanism and timing of CFL development are unknown; the participants were limited to older people because it was a cadaver study; the measurements were made on a surface specimen; and the limb position was not constant because the body was fixed. In future prospective investigations, studies on living patients should focus on lifestyle, foot pain, foot stability, and muscle strength.

## 5. Conclusions

In this study, the degeneration of the articular surface was mainly observed on the medial side and the CFL was wider in cases of degeneration. The degeneration of the talofibular joint surface can be screened by measuring the CFL width using an ultrasound imaging system. Further clinical studies utilizing MRI and ultrasound imaging devices are required. In the future, monitoring CFL width over time using ultrasound imaging devices may prove to be an effective method for the early detection of talar cartilage damage.

## Figures and Tables

**Table 1:** Distribution of taloarticular surface degeneration and its proportion.

Location	Proportion
Overall joint surface degeneration	40.0% (20/50 feet)
Medial degeneration	100% (20/20 feet)
Both (medial and lateral) degeneration	40.0% (8/20 feet)
Only medial degeneration	60.0% (12/20 feet)
Only lateral degeneration	0% (0/20 feet)

We found 20 of 50 feet exhibited joint degeneration, all of which showed medial degeneration; 8 of the 20 feet had medial and lateral degeneration, whereas 12 had only medial degeneration. No cases with only external degeneration were observed.

**Table 2:** Comparison between the Degeneration (+) and Degeneration (−).

		Degeneration (−) (N = 30)	Degeneration (+) (N = 20)	*p*
	1: TNL	19.95 ± 4.88	19.16 ± 5.29	0.589
Length (mm)	2: TSL	18.13 ± 5.83	16.52 ± 6.29	0.358
	3: TCL	16.80 ± 4.02	15.29 ± 4.59	0.225
	4: PTTL	12.64 ± 3.21	12.63 ± 4.01	0.719
	5: ATFL	16.92 ± 4.88	19.19 ± 4.69	0.108
	6: CFL	20.32 ± 5.09	20.45 ± 4.28	0.927
	1: TNL	10.53 ± 2.89	9.24 ± 2.71	0.121
Width (mm)	2: TSL	9.05 ± 2.41	8.10 ± 1.57	0.127
	3: TCL	10.01 ± 3.36	9.15 ± 2.05	0.311
	4: PTTL	9.03 ± 2.59	9.78 ± 2.95	0.347
	5: ATFL	20.70 ± 7.20	18.17 ± 7.15	0.228
	6: CFL	7.38 ± 1.57	9.74 ± 2.48	0.000 *
	1: TNL	0.82 ± 0.42	0.72 ± 0.37	0.401
Thickness (mm)	2: TSL	1.06 ± 0.46	0.83 ± 0.37	0.068
	3: TCL	1.05 ± 0.31	0.98 ± 0.54	0.524
	4: PTTL	1.01 ± 0.45	0.93 ± 0.37	0.512
	5: ATFL	1.45 ± 0.75	1.49 ± 0.54	0.836
	6: CFL	1.43 ± 0.49	1.40 ± 0.48	0.832

Data are presented as mean ± standard deviation (SD). Significant differences between the two groups were observed only in the CFL range. * *p* < 0.05. TNL, tibionavicular ligament; TSL, tibiospring ligament; TCL, tibiocalcaneal ligament; PTTL, superficial posterior tibiotalar ligament; ATFL, anterior talofibular ligament; CFL, calcaneofibular ligament.

**Table 3:** Area under the curve values.

Area Under Curve	SD	Asymptotic Significance Probability	Asymptotic 95% Confidence Interval	
			Lower Limit	Upper Limit
78.4%	0.074	0.001	63.9	93.0

## Data Availability

The data that support the findings of this study are available from the corresponding author upon reasonable request. Neither the patients nor the public were involved in the design, conduct, reporting, or dissemination plans of our research.
